# Design and methods of CYCLE-HD: improving cardiovascular health in patients with end stage renal disease using a structured programme of exercise: a randomised control trial

**DOI:** 10.1186/s12882-016-0294-7

**Published:** 2016-07-08

**Authors:** M. P. M. Graham-Brown, D. S. March, D. R. Churchward, H. M. L. Young, M. Dungey, S. Lloyd, N. J. Brunskill, A. C. Smith, G. P. McCann, J. O. Burton

**Affiliations:** John Walls Renal Unit, University Hospitals Leicester NHS Trust, Leicester, UK; Department of Infection Immunity and Inflammation, School of Medicine and Biological Sciences, University of Leicester, Leicester, LE1 9HN UK; Robertson Centre for Biostatistics University of Glasgow, Glasgow, UK; Department of Cardiovascular Sciences, University of Leicester and NIHR Leicester Cardiovascular Biomedical Research Unit, Glenfield Hospital Leicester, Leicester, UK; National Centre for Sport and Exercise Medicine, School of Sport, Exercise and Health Sciences, Loughborough University, Loughborough, UK

**Keywords:** Haemodialysis, Cardiovascular disease, Sudden cardiac death, Intra-dialytic cycling, Intra-dialytic exercise

## Abstract

**Background:**

There is emerging evidence that exercise training could positively impact several of the cardiovascular risk factors associated with sudden cardiac death amongst patients on haemodialysis. The primary aim of this study is to evaluate the effect of an intradialytic exercise programme on left ventricular mass.

**Method and design:**

Prospective, randomised cluster open-label blinded endpoint clinical trial in 130 patients with end stage renal disease on haemodialysis. Patients will be randomised 1:1 to either 1) minimum of 30 min continuous cycling thrice weekly during dialysis or 2) standard care. The primary outcome is change in left ventricular mass at 6 months, assessed by cardiac MRI (CMR). In order to detect a difference in LV mass of 15 g between groups at 80 % power, a sample size of 65 patients per group is required. Secondary outcome measures include abnormalities of cardiac rhythm, left ventricular volumes and ejection fraction, physical function measures, anthropometric measures, quality of life and markers of inflammation, with interim assessment for some measures at 3 months.

**Discussion:**

This study will test the hypothesis that an intradialytic programme of exercise leads to a regression in left ventricular mass, an important non-traditional cardiovascular risk factor in end stage renal disease. For the first time this will be assessed using CMR. We will also evaluate the efficacy, feasibility and safety of an intradialytic exercise programme using a number of secondary end-points. We anticipate that a positive outcome will lead to both an increased patient uptake into established intradialytic programmes and the development of new programmes nationally and internationally.

**Trial registration number:**

ISRCTN11299707 (registration date 5^th^ March 2015).

**Electronic supplementary material:**

The online version of this article (doi:10.1186/s12882-016-0294-7) contains supplementary material, which is available to authorized users.

## Background

Patients on haemodialysis (HD) have extremely high rates of cardiovascular disease (CVD) related mortality [1]. US renal data system (USRDS) data suggests it is the leading cause of mortality in prevalent HD patients, accounting for 42.3 % of all deaths [2]. There is good evidence that these excessive rates of CVD are driven by a different set of processes than in the general population and attempts to modify traditional cardiac risk factors have not improved outcomes in HD patients [3]. According to the USRDS database, up to 64 % of all cardiac mortality among HD patients is due to sudden cardiac death (SCD) or arrhythmias [4] of an order of around 100 times higher than the background population [5]. Classical atherosclerotic disease is the leading cause of myocardial ischaemia in the general population [6], but this is not the case in HD patients, who are subject to a unique set of factors that change the cardiac environment and lead to changes that alter cardiac structure and function [7]. These changes are associated with SCD among HD patients and include: abnormalities in myocardial structure and function such as left ventricular hypertrophy (LVH) which is present in 75 % of dialysis patients; interstitial fibrosis and microvascular disease; with chronic volume overload and large volume ultrafiltration during dialysis treatments also contributing to the excess burden of CVD observed [8–10]. The recurrent and frequent stresses on the heart from ultrafiltration are associated with an increase in ventricular arrhythmias [11] that associate with SCD and raised biomarkers of cardiac myocyte damage as well as being independent predictors of HD related cardiac injury [12] and ultimately myocardial fibrosis [13]. To date, efforts to address these changes and improve outcomes have concentrated on medical therapies (e.g. pharmaceutical agents and implantable cardiac defibrillators) which are yet to show positive benefits [14] and studies have also demonstrated significantly increased mortality rates in HD patients after coronary revascularisation compared to the general population confirming the differences in pathogenesis [15], and again highlighting the limitations of current treatment options for HD patients.

### The benefits of exercise for patients on haemodialysis

Exercise is not as commonly-used a therapeutic intervention in HD patients as it is in other chronic diseases, e.g. cardiac and respiratory patients, and although it is clear there are a number of potential benefits from exercise in this patient population the quality of evidence is variable and there are large gaps in the evidence base. There are several systematic reviews that summarize the potential cardiovascular benefits of exercise in HD patients, as well as the likely benefits to dialysis quality, quality of life and other health related benefits [16, 17]. Exercise interventions have been largely divided into those that occur between dialysis sessions (interdialytic exercise) and those that occur during dialysis (intradialytic exercise). Whilst there is evidence that interdialytic training may yield superior cardio-respiratory adaptations, there is also a much a higher drop-out rate from such programmes [18, 19]. Intra-dialytic exercise programmes are associated with significant improvements in cardio-respiratory reserve compared to control patients and have very good adherence rates [18].

### Cardiovascular disease, exercise and HD patients

In the general population, lifestyle changes that result in increased physical exercise lower mortality [20]. Unfortunately, HD patients are less active than even sedentary healthy people with <50 % of HD patients reporting exercising once a week and unsurprisingly, higher mortality rates have been shown in such patients [21]. Exercise training during or outside of dialysis has been shown in a number of uncontrolled and non-randomised trials to lead to significant improvements in a number of cardiovascular risk factors that predispose to SCD, both traditional and those unique to patients with end stage renal disease on HD [22–24]. These studies, however, are all limited by either small sample size, non-randomised or uncontrolled design and there are no large studies that have used cardiac MRI (CMR) to assess changes in myocardial structure and function in HD patients who undergo a structured programme of exercise.

### CMR in HD patients

The term uraemic cardiomyopathy has traditionally been given to a constellation of changes in cardiac structure and function seen in patients with end stage renal disease (ESRD) that include: left ventricular hypertrophy, left ventricular dilatation and left ventricular systolic dysfunction. All of these structural and functional changes have been shown to associate with poor cardiovascular outcomes [25, 26]. It is acknowledged that studies which have used echocardiography have limited accuracy and reproducibility in defining geometric parameters and indices of systolic and diastolic function. This may be especially true in HD patients who are subject to significant changes in cardiac filling from fluid status [27]; indeed LV mass and cavity size may be overestimated in up to 50 % of dialysis patients [28]. CMR has been shown in HD patients to be a reliable technique for LV mass measurement, with excellent intra- and inter-observer variability for end-diastolic volume, end-systolic volume and LV mass [29]. LV mass is a proven continuous variable in a graded relationship with cardiovascular risk [30], and cardiovascular outcomes improve as LV mass regresses, underlying the importance of being able to accurately quantify LV mass change in intervention studies.

Echocardiography gives only limited information about tissue characterisation, compared to CMR. The risk of nephrogenic systemic fibrosis currently precludes the administration of gadolium-based contrast agents to HD patients [31]. New native T1 mapping techniques have been shown to correlate very well with histological collagen percentage in patients with severe aortic stenosis [32] and native T1 mapping has been shown to be reproducible in patients with Fabry’s disease and amyloidosis [33, 34]. Native T1 mapping holds great promise in further defining pathogenesis and tissue characterisation in HD patients and patients with ESRD and CKD.

Whilst atheroma related arterial disease remains an important factor in patients on HD, arteriosclerosis is of at least equal importance. Arteriosclerosis is a process characterised by hypertrophy and increased collagen deposition in the medial layer of the arterial wall, with circumferential calcification, and commonly occurs in patients with ESRD and CKD [35]. This causes arterial stiffness and has been shown to independently predict cardiovascular morbidity and mortality in ESRD [36]. There is a significant amount of research examining the relationship between arterial stiffness and cardiovascular disease in patients with ESRD and CKD, with much of it derived from applanation tonometry techniques that measure aortic pulse wave velocity and measures of aortic/arterial distensibility [36–38]. More recently CMR has been used to assess aortic distensibilty and there is gathering evidence of both the importance of aortic distensibility and its relationship with the development of uraemic cardiomyopathy, as well as the validity of using CMR for its assessment [39–41].

Myocardial strain and strain rate have been shown to be early markers of contractile dysfunction in many conditions that precede declines in ejection fraction. Systolic strain and strain rates have traditionally been assessed with CMR using tissue tagging techniques [42]. Increased circumferential basal strain and strain rates and reduced longitudinal function may occur in non-diabetic CKD patients, before any other structural or functional changes are apparent; suggesting it may be a very early indicator of uraemic cardiomyopathy [43]. Whilst tissue tagging is proven to be reproducible, scan acquisition requires additional long breath-holds and analysis can be cumbersome. Newer methods of strain and strain rate analysis are now available that can assess LV strain and strain rates directly from cine images. Our group has shown that Feature Tracking has excellent reproducibility and maybe more robust than tissue tagging in acute myocardial infarction patients [44, 45]. There are currently no studies that have used CMR to assess the effects of exercise on cardiac structure and function in HD patients.

The primary aim of this study is to investigate the effects of a six month programme of intra-dialytic exercise on LV mass as assessed by CMR. We will assess other cardiac structural and functional end-points, as well as biochemical markers of acute and chronic cardiac dysfunction, anthropometric measurements and measures of physical function and quality of life. An important secondary aim is to establish whether intradialytic exercise training is associated with an increase in cardiac arrhythmia, thereby addressing one of the major safety concerns.

## Methods

### Design

The protocols described in this manuscript are quorate with the most recent study protocol for the CYCLE-HD trial (version 3 15/05/2015). This study is a prospective, randomised, open-label, blinded endpoint (PROBE) study, with cluster design. The trial was given ethical approval by the NHS Research Ethics Committee East Midlands (Northampton; REC ref: 14/EM/1190). We aim to recruit 130 patients with established renal failure on maintenance HD. The study will initially take place at three dialysis units within the East Midlands Renal Network with the provision of an intradialytic cycling programme being randomised depending upon shift pattern. This study is a fully funded National Institute for Health Research (NIHR) portfolio study ‘CYCLE-HD’ (UKCRNID 17951) and is registered with the ISCRTN registry (ISRCTN11299707).

### Randomisation

Current practice in UK dialysis centres dictates that patients dialyse in one of two cohorts, either on a Monday, Wednesday and Friday or Tuesday, Thursday and Saturday. In this study, each dialysis shift will be randomised to either continue on standard dialysis therapy (control group) (see ‘Usual Care: Haemodialysis’ below) or standard dialysis therapy plus the intervention of intradialytic exercise (exercise group). This method of randomisation was modelled by the Robertson Biostatistics Centre at the Glasgow Clinical Trials Unit and peer reviewed by the National Institute of Health Research (NIHR).

### Aims of the study

- To assess the effects of a six-month intra-dialytic programme of exercise on cardiovascular structure and function
- To assess the effects of a six-month intra-dialytic programme of exercise on changes in biomarkers of cardiovascular disease and systemic inflammation, physical function, body composition and quality of life measures

Two sub-studies will:

- Assess the short-term arrhythmogenic potential of intradialytic exercise
- Assess the reproducibility of a number of CMR parameters not previously assessed or validated in HD patients

### Primary hypothesis

- A six month programme of intradialytic exercise will lead to a regression in LV mass in patients receiving maintenance HD

### Secondary hypotheses

- A six-month intradialytic programme of exercise for HD patients is safe with no increase in either cardiac arrhythmias or fibrosis
- A six-month intradialytic programme of exercise leads to an improvement in CV indices associated with an increased CV risk and SCD including: LV function; myocardial fibrosis; LV strain; cardiac arrhythmias; autonomic dysfunction; raised biochemical markers of cardiac damage and heart failure and; systemic inflammation
- A six-month intradialytic programme of exercise leads to an improvement in physical function and QOL

### Outcome measures

Primary outcomes measure:

1. LV mass in grams using CMR

Secondary outcome measures:

1. Cardiac arrhythmias, including frequency; isolated ectopy as a percentage of the total beats on the Holter monitor record; ventricular arrhythmias stratified according to the Lown classification with classes 3 and above taken as complex; and heart rate variability. Patients undergoing exercise intervention will undergo additional 48 h recording to assess rhythm data, during and after exercise on dialysis. Interim analysis is planned to identify any potential adverse rhythm disturbances associated with exercise
2. Left ventricular end-diastolic and end-systolic volumes and ejection fraction (%) using CMR and echocardiography. Measures of systolic and diastolic dysfunction and myocardial fibrosis using CMR and ECHO
3. Aortic stiffness: Aortic distensibility and/or pulse wave velocity
4. Anthropometric measures including, but not limited to, weight, height and waist circumference
5. Physical function assessed by shuttle walk tests, sit to stand tests, balance tests and gait speed (the short physical performance battery)
6. Objective assessments of physical activity using validated questionnaires and tri-axial accelerometry
7. Quality of life (QOL) using validated questionnaires
8. Blood markers of inflammation, cardiovascular dysfunction and cardiovascular risk
9. Selected clinical episodes including: all hospitalisations; all-cause mortality; cardiovascular mortality; and cardiovascular morbidity including non-fatal myocardial infarction, cerebrovascular event, critical limb ischaemia

### Participant identification and recruitment

Prevalent adult patients undergoing in-centre maintenance HD at one of the enrolled units are eligible for inclusion in the study. Full lists of inclusion and exclusion criteria are included in Table 1.

**Table 1:** Eligibility and Exclusion criteria

Eligibility Criteria	Exclusion Criteria
• Prevalent HD patient (> three months) • Aged 18 years or older • Able and willing to give informed consent	• unable to participate in current exercise programme due to perceived physical or psychological barriers • unable to undergo MRI scanning (metal implants, severe claustrophobia) • unfit to undertake exercise according to the American College of Sports Medicine (ACSM) guidelines

Patients will be recruited from centres within the East Midlands Renal Network, which cares for approximately 820 HD patients across four Counties in the United Kingdom. The three principal centres in Leicestershire care for around 350 patients. We anticipate 80 % will meet the inclusion criteria (320 patients). Conservatively assuming a 50 % consent/participation rate this will leave approximately 160 patients from which we will need to recruit 130.

### Consent

Consent will be performed according to the rules of good clinical practice. The cluster randomisation design of this trial means that at recruitment researchers will know, based on the days on which they have dialysis, whether the patient will be in the control or the intervention group. Although it will become obvious to patients when they commence the trial whether they will be in the control group, or the intervention group, this will not be explained to patients before they consent to reduce selection bias. A copy of the consent form is included as Additional file 1.

#### Data collection

An electronic case report form (eCRF) will be used to collect all study data. Only authorised personnel will be able to make entries, amendments or changes to patient data on the eCRF. The trial dataset will be held and analysed by the Glasgow CTU. Investigators will not have access to the full dataset until the trial is closed.

### Study timeline

#### Baseline assessments

**Fig. 1:**
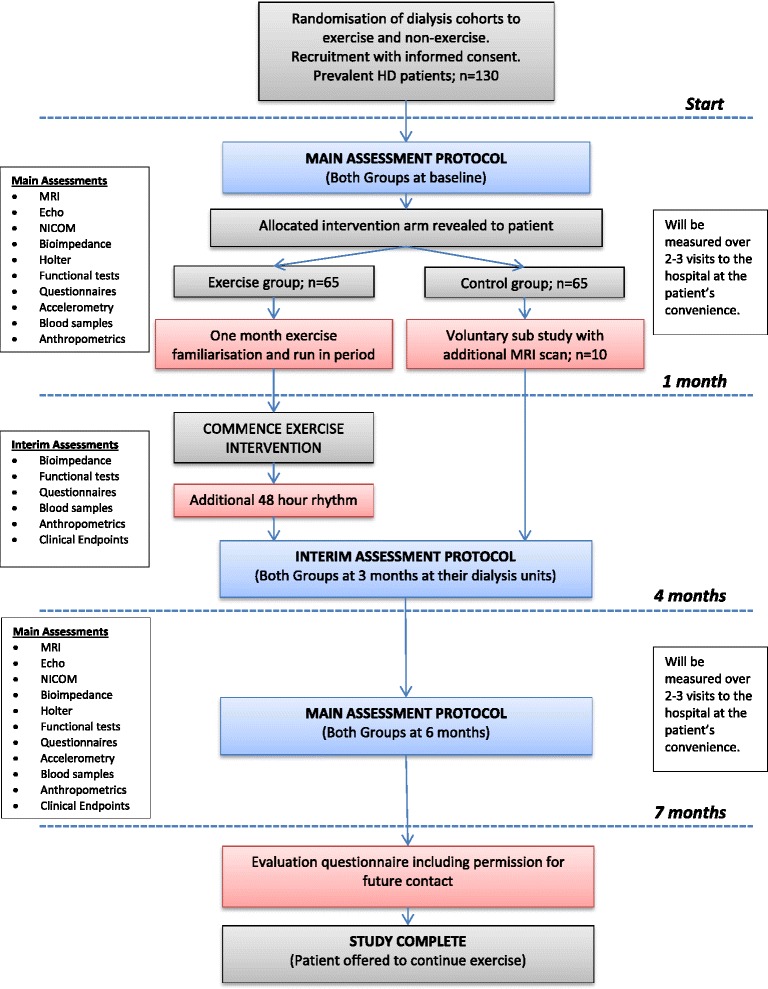
CYCLE HD study protocol timeline

The CYCLE-HD study protocol timeline is shown in Fig. 1. Baseline assessments will be undertaken on a non-dialysis day at the NIHR-Leicester Cardiovascular BRU.

#### Cardiac MRI

**Fig. 2:**
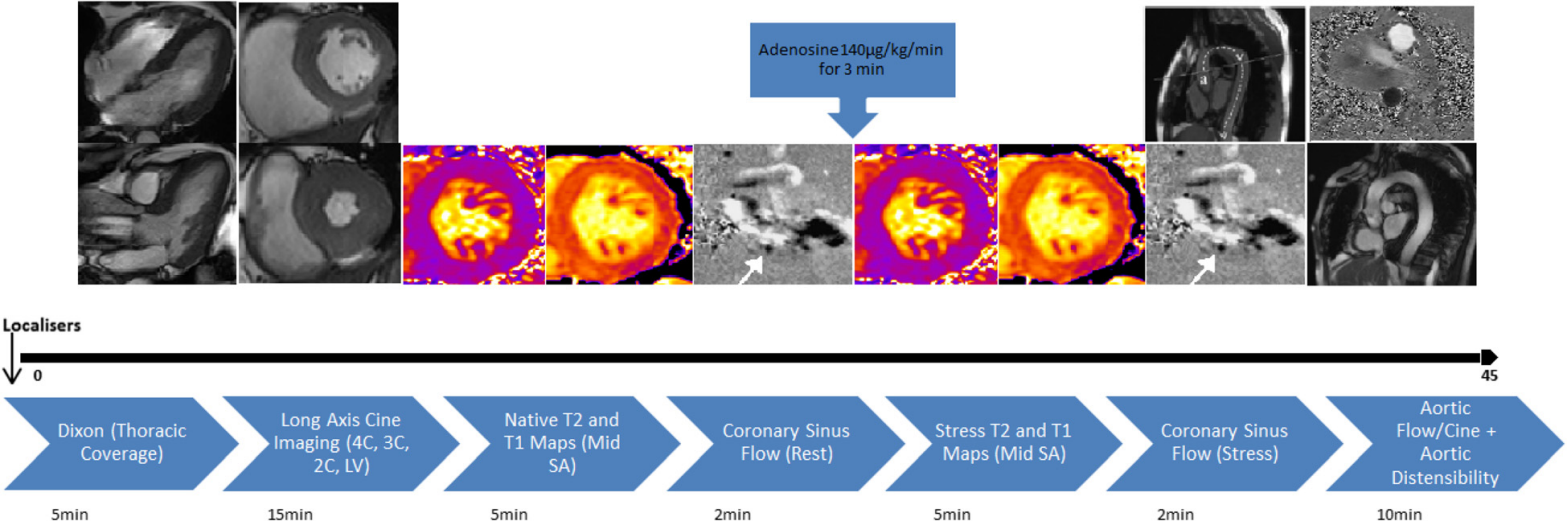
Cardiac MRI Protocol. (4/3/2C, 4/3/2 Chamber; LV, Left Ventricular; SA, Short Axis)

The CMR scan protocol timeline in shown in Fig. 2. Patients will be imaged on a 3 Tesla (3 T) CMR platform (Skyra, Siemens Medical Imaging, Erlangen, Germany). The CMR protocol will be similar to that previously described but without contrast administration [46]. LV volumes and mass will be quantified with epicardial and endocardial contours of a contiguous stack of multiphase ventricular short axis cines (10–12 slices, 8 mm slice thickness, 25 % gap) at end-diastole and end-systole. Native T1 and T2 mapping at mid-ventricular level will be undertaken for tissue characterisation, with non-contrast T1 mapping offering the opportunity to assess myocardial fibrosis [47, 48]. Vasodilator stress with adenosine at an initial dose of 140 μg/kg/min for three minutes will be given and myocardial perfusion will be assessed with the response in signal intensity on T1 and T2 maps compared to baseline [49]. Adequate haemodynamic response is assessed by either ≥10 % heart rate increase or ≥10 mmHg decrease in systolic blood pressure. Adenosine dose may be increased incrementally to an upper limit of 210 μg/kg/min to achieve haemodynamic response if needed. Feature tracking and/or tissue tracking will be used to assess systolic strain and diastolic strain rate [44]. Myocardial perfusion reserve (the ratio of global myocardial blood flow at rest versus stress) will be assessed using phase contrast velocity mapping of the coronary sinus before and after adenosine stress [50]. Aortic compliance will be assessed by measuring distensibility of the ascending and descending thoracic aorta. Changes in the cross-sectional luminal area of aorta in a 5 mm thick slice will be measured and concomitant measurement of blood pressure at the time of sequence acquisition will allow calculation of aortic distensibilty and pulse wave velocity [41]. Three point Dixon images for quantification of thoracic visceral fat content will be acquired in patients who tolerate the scan well.

#### Echocardiography

Patients will be scanned by an accredited sonographer on a Phillips iE33 platform (Best, The Netherlands) in the NIHR-Leicester Cardiovascular BRU. Assessments will include: LV size and function; LV mass; relative wall thickness and geometry as per the American society of echocardiography guidelines [47]. In addition specific focus will be paid to diastolic dysfunction and end-diastolic integrated backscatter measurements that are directly related to the presence of myocardial fibrosis and validated in HD patients [51–55]. These data are key to assessing the long term effect of exercise on myocardial fibrosis in HD patients as the use of late gadolinium enhancement on CMR is not possible due to the risk of nephrogenic systemic fibrosis [56]. Speckle tracking for strain assessment will be also be undertaken.

#### Holter monitor

Patients will undertake 48 h Holter monitoring (Schiller, medilog®AR12 plus/AR4 plus/FD5 plus, Baar, Switzerland) that will start before dialysis and terminate just before the subsequent dialysis treatment 48 h later. In a sub-study, the intervention group will undergo a further 48 h Holter recording that will start before a dialysis session and includes the cycling exercise intervention and continues for the same time period afterwards. This will enable interim analysis to ensure that there are no adverse rhythm disturbances associated with exercise in the intervention group.

#### Non-Invasive Cardiac Output Monitor (NICOM)

Patients will have cardiovascular parameters measured by bioreactance prior to dialysis, using the NICOM (Cheetah Medical, Maidenhead, UK) in-line with their guidelines for use. This device assesses phase shifts in transthoracic voltage when a high frequency current is applied across the torso [57]. From this validated technique [58, 59] the NICOM provides readings of cardiac output, stroke volume, heart rate, total peripheral resistance, cardiac power and ventricular ejection time.

#### Blood sampling

Blood samples will be collected from the arterial needle before dialysis. Thirty millilitres will be collected to be centrifugated at 20 °C at 2500 × *g* for 15 min. Plasma will then be pipetted into cryotubes and frozen at -80 °C in an electronically monitored freezer for analysis in batches throughout the study. All samples will be collected, stored and disposed of in accordance with the Codes of Practice as laid out by the Human Tissue Authority.

#### Physical function tests

Patient physical functioning will be assessed by the shuttle walk tests, the sit-to-stand-60 (STS60) test and the short physical performance battery, all of which have been used extensively as accurate measures of aerobic capacity, lower leg strength and physical function in HD patients [60–62].

#### Anthropometric measures

Patients will have measures of hip and waist circumferences, body weight and body composition. Body composition will be analysed prior to dialysis, using bioimpedance spectroscopy (BIS). For this we will use the Body Composition Monitor (BCM Fresenius Medical Care, Bad Homburg, Germany), validated for use in HD patients [63], allowing for interpretation of normally hydrated lean and adipose tissues as well as excess fluid [64, 65] and other variables.

#### Quality of life and physical function

Patients will complete the following questionnaires to capture data about physical activity and quality of life: Short form-12 version 2 (SF-12) which has physical and mental component scores; the EQ-5D-5L which provides a functional index value and visual analogue score; the Palliative Outcome Scale–Symptoms Renal (POS-S Renal) to assess symptom burden; The Hospital Anxiety and Depression Scale (HADS) to assess anxiety and depression levels; The Leicester Dialysis Patients–Physical Activity Questionnaire (LDP-PAQ), which assesses perceived physical activity, perceived stage of change, perceived self-efficacy, self-reported physical activity levels and perceived barriers to exercise participation. Objective data on patient physical activity levels will also be gained from tri-axial accelerometers (Sensewear; BodyMedia, Inc. Pittsburgh, PA), which will calculate steps taken, energy expenditure (kcALs) and average metabolic equivalents (METS).

#### Collection of routine clinical information

As part of routine clinical care, a number of parameters will be collected on a monthly basis. These include (but are not limited to) blood pressure, weight and fluid removal for every dialysis session as well as biochemical and haematological blood tests.

#### Follow-up assessments

Follow up visits are summarised in Fig. 1. A one-month run-in period allows the exercise group to familiarise themselves with intradialytic cycling. Three months after this run in period (four months from baseline), interim assessments will be conducted. This will involve all baseline assessments except the CMR, echocardiogram and NICOM. The control group will also undergo interim assessments at month four.

Final assessments will be conducted for both exercise and control groups after seven months. Assessments at study completion will be identical to the baseline visit.

Following study completion, patients will be offered the opportunity to continue with intradialytic cycling if they wish and control patients will be offered the chance to move to a shift where exercise intervention is available. We will also ask patients to complete a feedback and evaluation form for ongoing development of study and future service provision.

#### Sub-study

Additional informed consent will be sought after the first CMR scan for ten control patients to be re-scanned using an identical scan protocol to assess inter and intra-observer variability and reproducibility of all scan parameters.

#### Investigation reporting

Cardiac imaging will not be reported until after the patient has completed the study so as not to influence the study outcome. However life-threatening incidental findings identified during scan acquisition will be reported to the chief investigator, the data safety management board (DSMB) and the sponsor, and a full report will be issued to aid clinical care and ensure patient safety.

### Image analysis

All scans will be anonymised and analysed off-line blinded to patients’ data and treatment arm. CMR analysis will be both visual and quantitative following international recommendations [66]. Quantitative analysis will be performed by a single operator using FDA approved commercially available software. This will include: LV mass(g); End-diastolic volume (ml); end-systolic volume (ml); stroke volume (ml); and ejection fraction (%). All volumetric data will be indexed to body surface area.

T1 and T2 values will be taken from a mid-short axis slice of the LV by drawing epicardial and endocardial borders [49]. LV strain and strain rate will be assessed as previously described [44]. Endocardial and epicardial contours will be manually drawn onto the end-diastolic image and propagated and LV endocardial and epicardial circumferential and longitudinal strain and strain rates will be calculated.

Aortic distensibility will be analysed as previously described [67]. The ascending thoracic aortic area will be manually identified as a region of interest using JIM version 6 (Xinapse software, UK) and graphically represented against time. Aortic distensibility will be determined using the validated formula:

$$ \left( maximum\  aortic\  area- minimum\  aortic\  area\right)/\left( minimum\  aortic\  area\kern0.5em X\kern0.5em \varDelta P\right) $$

ΔP is the brachial pulse pressure reading performed during CMR [68].

#### ‘Usual care’: haemodialysis

Haemodialysis is a form of renal replacement therapy that replaces part of the excretory function of the kidneys by filtering waste, removing fluid and restoring electrolyte and acid-base balance by transferring blood from the body through a dialysis machine and returning it to the body. ‘Adequate’ dialysis is often assessed by calculating the clearance of small molecules during a dialysis session and guidelines for minimum suggested clearance exist [69]. This is only one measure of dialysis ‘adequacy’ however, and a dialysis machine only replaces certain aspects of renal function and patients receive a number of additional interventions as part of their ‘usual’ dialysis care. There are defined treatment targets for blood pressure, calcium, phosphate, haemoglobin and iron stores – including management of anaemia with erythropoietin stimulating agents (ESAs) and intravenous iron infusions [69] in addition to measures of adequacy from small molecule clearance. Both trial arms will continue ‘usual care’ aimed at achieving these targets to allow assessment of the effects of intradialytic exercise in addition to usual dialysis care.

#### Intervention: intradialytic exercise training

**Fig. 3:**
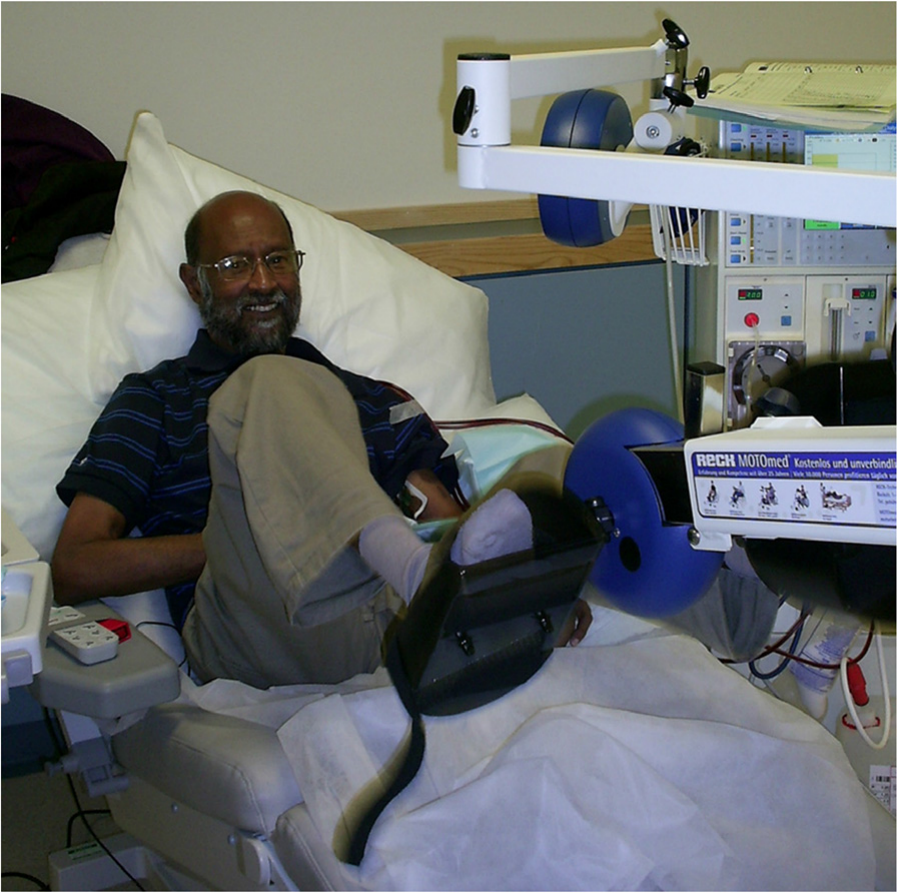
Patient undertaking intradialytic cycling on specially adapted cycle

The intervention is a 6-month progressive intradialytic cycling programme. The intervention group will use specially adapted and calibrated exercise cycles (Letto series; Motomed, Reck, Germany, see Fig. 3) three times a week during dialysis, aiming for 30 min continuous cycling at a Rating of Perceived Exertion (RPE) of 12–14 [70] adjusting resistance as required to progress training (Fig. 3). Our pilot exercise programme of 31 patients has been well tolerated with no adverse symptoms reported in any of the exercising patients. It is accessible to patients of different ages, gender, cultures and ethnicities.

Progressive training will be allowed for patients unable to complete 30 min continuous cycling, until this target is achieved and patients will be allowed to complete longer exercise bouts if they request. This individualised style of exercise programme has been guided by patient feedback during studies we have previously conducted [71]. There will also be a run in period of one month, again as a result of patient feedback, to enable individuals to get used to the equipment, to build confidence and to ensure they will be able to achieve the required exercise intensity to derive a benefit before the 6 months of ‘formal’ training begins. Patients will be regularly visited throughout the period of study to ensure compliance and also progression using a specially designed RPE-guided incremental exercise test to confirm appropriate exercise intensity.

### Sample size calculation

A study by London *et al.* showed a 10 % decrease in LV mass (≈29 g) translated into a 28 % decrease in mortality risk from cardiovascular causes over a five-year follow-up of a cohort of HD patients (a 1 g decrease translated to a 1 % decrease in CV mortality risk) [48]. A previous study of exercise in HD patients has shown a reduction in left ventricular mass index and increase in left ventricular ejection fraction (LVEF) assessed by echocardiogram, but not to significance [72]. In a study of the benefits of exercise training in 11 hypertensive elderly patients, 6 months of exercise (walking, jogging or cycling training) produced a 15 g difference in LV mass between the intervention group and controls [73]. CMR data from an RCT that assessed the benefits of frequent HD on LV mass in HD patients [74] provides an estimate for the standard deviation (SD) for change in LV mass of 25.9 g for the control group; we will assume that the SDs are similar in both groups. A difference (of change from baseline) between the two groups in LV mass of 15 g is deemed to be clinically significant. We have assumed an intra-cluster correlation coefficient of 0.02. From previous pilot work [75] we expect a drop-out rate of 10 % from exercise intervention. To have 80 % power to detect a difference between treatment groups of 15 g, with group standard deviations of 25.9 g, 65 patients are required in each group, accounting for a 10 % study attrition rate.

#### Statistical analytic approach for primary outcome

The primary outcome is the change from baseline at six months in LV mass. Change from baseline will be calculated for each subject as the value at month six minus the baseline value. Differences in primary outcome measure between the intervention groups (exercise and control) will be tested within a linear mixed model containing a covariate for the intervention group and a random effect for the cohort (centre and shift). These models may be further adjusted for any imbalances between the intervention groups with respect to the baseline characteristics. All statistical analyses will be performed by a biostatistician from the Robertson Centre for Biostatistics, University of Glasgow.

### Safety reporting

Due to the nature of ESRD and of HD, patients are likely to experience adverse events throughout the course of the study. Patients on HD have a large burden of co-morbid disease and, acute illness resulting in hospitalisations, new medical problems and deterioration of existing medical problems are expected throughout the study period.

All adverse events (AEs) or adverse reactions (ARs) and serious adverse events (SAEs) or serious adverse reactions (SARs) will be recorded from the time a patient enters the study to the final study visit. Each AE or AR will be considered for severity, causality and expectedness and may be reclassified as an SAE or SAR depending on the circumstances.

An SAE is any AE that:

- is life threatening
- requires hospitalization or prolongation of a hospital admission
- results in a persistent or significant disability or incapacity
- is a congenital anomaly
- results in death

All unexpected SAEs will be reported to the Glasgow Clinical Trials unit (CTU) within 7 days of awareness of the event, including a report assessing event intensity and likelihood of causality (see below) from the chief investigator or suitable nominated investigator. Study investigators will report all unexpected AEs, ARs, SAEs and SARs to the clinical trials unit, the sponsor, the Research Ethics Committee, and the Data Safety Management Board (DSMB).

Unexpected non-serious AEs will be assessed by the chief investigator, and should include an assessment of intensity and causality (see below), with reports being made within 14 days. These will be reported to the CTU and if appropriate to the sponsor, the Research Ethics Committee, and the DSMB.

The following guidance will be used to assess the intensity of an AE or an AR:

- Mild: The patient is aware of the event or symptom, but it can tolerate it easily
- Moderate: The patient experiences sufficient discomfort to interfere with or reduce his or her usual activities
- Severe: Functional levels are significantly impaired by the event such that patients can no longer carry out usual activities, or life is at risk from the event

The following guidance will be used to assess causality between an AE or AR and study participation:

- Unrelated: A causal relationship can definitely be excluded as another documented cause of the AE/AR is most plausible
- Unlikely: A causal relationship is improbable and another documented cause of the AE/AR is most plausible
- Possible: A causal relationship is clinically/biologically plausible and there is a plausible time sequence between AE/AR and study participation
- Probable: A causal relationship is clinically/biologically highly plausible and there is a plausible time sequence between onset of the AE/AR and study participation

### Data safety management board

The DSMB will be an independent group of experts, consisting a nephrologist, a cardiologist, lay member and a statistician, who will monitor patient safety and treatment efficacy data while the clinical trial is ongoing; the primary mandate of this committee is to protect patient safety. If adverse events of a particularly serious type are more common in the experimental arm compared to the control arm, then it would be within the remit of the DSMB to consider termination of the study if the risks outweigh the benefits for patients. All SAEs deemed to have a causal relationship with the intervention will be reviewed by the DSMB for individual consideration of patient safety and continuation within the trial.

#### Intervention fidelity and monitoring

Experience to date with our unit based exercise programme has shown this programme to work with very small dropout rates of <5 % and a number of studies of similar size with exercise interventions of 6–10 months report very good compliance (up to 99 %) with comparable dropout rates [71, 72]. Temporary noncompliance (e.g. intercurrent illness, holiday) will be allowed for by adding missed exercise sessions onto the end of the study period. Apart from the exercise intervention in the study group, usual care will be continued for both groups. The involvement of the nursing staff with the study will help aid compliance and monitoring and there will be frequent contact between patients and research staff.

## Discussion

The primary aim of this study is to assess the effect of a six month programme of intradialytic exercise on LV mass as assessed by CMR. The cluster randomization design was chosen for the advantages in reducing exercise contamination of the control group and to ensure demographic homogeneity. There is the possibility that this could create selection bias if patients are aware which cohorts have been randomised to intervention and control before they are enrolled, but this will be minimised by not revealing the method of randomisation to patients until after they have consented to participation *and* completed all baseline assessments. This study will provide the opportunity to evaluate the effects of a structured programme of intradialytic exercise on a number of secondary outcomes. Whilst clinical outcome data will be collected, this study is not designed to evaluate the effects of exercise on mortality.

CMR used in this study is the gold standard technique for the quantification of LV mass, volumes and function. We anticipate it will be technically challenging to obtain high quality images for some patients who may struggle to lie flat for long periods or who find breath holding difficult. The CMR protocol has been carefully designed to acquire all desired images in the most efficient way possible and have pioneered techniques that allow shorter scan times, with the primary outcome measure obtained early in case of premature scan termination. The CMR radiographers at the NIHR-Leicester Cardiovascular BRU have many years’ experience obtaining scans from patients with similar burdens of co-morbid disease [45, 76, 77]. The increased signal noise ratio obtained at 3 T allows the faster acquisition of cine images (4–5 s) for patients who are poor at breath-holding and free-breathing techniques can also be used with acceptable image quality.

There are some data suggesting interdialytic exercise may yield superior cardio-respiratory adaptations to intradialytic exercise programmes, but with significantly higher drop-out rates [18, 19]. Given the proven benefits of intradialytic exercise programmes compared to control patients and the superior adherence rates [18], pragmatically an intradialytic exercise programme was chosen as it is the most likely to be translated successfully into clinical practice.

The adoption of intradialytic exercise programmes into clinical practice has been slow and this has, in part, been attributed to patient and clinician concerns regarding safety [78–82]. The study is designed so that interim analysis will provide data on the arrhythmogenic potential of intradialytic exercise. This should in turn help to alleviate patient and staff concerns regarding the safety of exercise on dialysis.

## Conclusion

This study will test the hypothesis that an intradialytic programme of exercise leads to a regression in left ventricular mass, an important non-traditional cardiovascular risk factor in end stage renal disease. We will also evaluate the efficacy, feasibility and safety of an intradialytic exercise programme using a number of secondary end-points. We anticipate that a positive outcome will lead to both an increased patient uptake into established intradialytic programmes and the development of new programmes nationally and internationally.

## Abbreviations

ACSM, American College of Sports and Exercise Medicine; BCM, body composition monitor; BIS, bioimpedence spectroscopy; CMR, Cardiac Magnetic Resonance Imaging; CVD, cardiovascular disease; DSMB, data safety management board; ECHO, echocardiogram; eCRF, electronic case report form; EQ-5D-5L, Euro Qol-5 Dimension-5 Levels; HD, haemodialysis; LV, left ventricular; LVEF, left ventricular ejection fraction; LVH, left ventricular hypertrophy; MRI, magnetic resonance imaging; NICOM, non-invasive cardiac output monitoring; NIHR, National Institute for Health Research; RCT, randomised-controlled trial; RPE, rating of perceived exercise; SCD, sudden cardiac death; SD, standard deviation; SF-12, short form-12 questionnaire

## Additional file

Additional file 1: Copy of the CYCLE-HD consent form. (DOC 93 kb)
