# Supplementary material for: Design and methods of CYCLE-HD: improving cardiovascular health in patients with end stage renal disease using a structured programme of exercise: a randomised control trial
Source: BMC Nephrol. 2016 Jul 8;17:69. doi: 10.1186/s12882-016-0294-7 (PMC4938939; doi:10.1186/s12882-016-0294-7)
Supplement: Additional file 1: — Copy of the CYCLE-HD consent form. (DOC 93 kb) [file 12882_2016_294_MOESM1_ESM.doc]

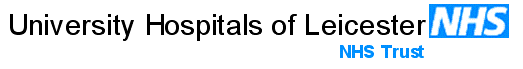


**Leicester Kidney Exercise Team**

**LEICESTER GENERAL HOSPITAL**

University of Leicester Academic Unit

Gwendolen Road, Leicester, LE4 5PW

0116 2584346

Centre Number:

Study Number: UHL 164347

Participant Identification Number for this trial:

**CONSENT FORM**

Title of Project: Improving cardiovascular outcomes in haemodialysis patients using a structured programme of exercise (CYCLE-HD). Please initial box

Name of Researcher: Dr James Burton YES / NO

- - - 1. I confirm that I have read the information sheet dated 15^th^ May 2015 (version 3) for the above study. I have had the opportunity to consider the information, ask questions and have had these answered satisfactorily.
      2. I understand that my participation is voluntary and that I am free to withdraw at any time without giving any reason, without my medical care or legal rights being affected.
      3. I understand that my contact details will be made available to the research staff involved in this project so that they can contact me to arrange details of my research study appointments, and I give permission for them to do so.
      4. I understand that a researcher will access my medical records to extract clinical information relevant to this study
      5. I understand that my General Practitioner will be informed of my taking part in this study.
      6. I understand that the samples collected as part of the research will be stored for 5 years in research laboratories at the University of Leicester to be used in laboratory tests.
      7. I understand that any tests carried out on the samples collected are part of a research programme and any results will not be used as a basis for diagnosis or treatment, either now or in the future.
      8. I understand that relevant sections of my medical notes and data collected during the study may be looked at by individuals from regulatory authorities, the Sponsor or from the NHS Trust, where it is relevant to my taking part in this research. I give permission for these individuals to have access to my records.
      9. If I withdraw from the CYCLE-HD study before the end, I consent that the samples collected will continue to be used in the research.
      10. I consent to stored samples being used in future research studies with national and local ethical approval.
      11. I consent to my contact details being kept on file for the purpose of being contacted about future research.
      12. I agree to take part in the above study.

Name of participant Date Signature

Name of person taking consent Date Signature
